# Subseafloor life and its biogeochemical impacts

**DOI:** 10.1038/s41467-019-11450-z

**Published:** 2019-08-06

**Authors:** Steven D’Hondt, Robert Pockalny, Victoria M. Fulfer, Arthur J. Spivack

**Affiliations:** Graduate School of Oceanography, University of Rhode Island Narragansett Bay Campus, 215 South Ferry Road, Rhode Island, 02882 USA

**Keywords:** Marine chemistry, Element cycles, Microbial ecology

## Abstract

Subseafloor microbial activities are central to Earth’s biogeochemical cycles. They control Earth’s surface oxidation and major aspects of ocean chemistry. They affect climate on long timescales and play major roles in forming and destroying economic resources. In this review, we evaluate present understanding of subseafloor microbes and their activities, identify research gaps, and recommend approaches to filling those gaps. Our synthesis suggests that chemical diffusion rates and reaction affinities play a primary role in controlling rates of subseafloor activities. Fundamental aspects of subseafloor communities, including features that enable their persistence at low catabolic rates for millions of years, remain unknown.

## Abstract (web summary)

Subseafloor microbial activities are central to global biogeochemical cycles, affecting Earth’s surface oxidation, ocean chemistry, and climate. Here the authors review present understanding of subseafloor microbes and their activities, identify research gaps, and recommend approaches to fill those gaps.

## Introduction

Subseafloor ecosystems constitute a significant portion of Earth’s biosphere. The estimated total number of cells in marine sediment (~10^29^)^1^ rivals estimated totals in the ocean and in soil^2^. Although total cell abundance is unknown for the igneous aquifer that underlies marine sediment, microbes are routinely recovered from this aquifer^3^, chemical traces of their activity are pervasive in its altered rock^4,5^ and formation fluid^6^, and physical textures suggestive of microbial alteration are common^7^. The total volume of subseafloor habitats is immense; the volumes of marine sediment and igneous basement cooler than 122 °C [the currently accepted high-temperature limit to life^8^] are respectively 2.6 × 10^8^ km^3^ ^9^ and 1.0 × 10^9^ km^3^ (Methods). The volumes of potentially habitable void space within the sediment^9^ and basement, respectively, are about 8.5 × 10^7^ km^3^ and 8.0 × 10^7^ km^3^ (each equal to about 6% of ocean volume [1.3 × 10^9^ km^3^]^10^).

Mean respiration per cell is very low in subseafloor sediment^11,12^. Although their total abundance is high, microbial cells in marine sediment are generally much smaller than microbial cells in the surface world. Consequently, the total biomass of marine sedimentary life is <1% of Earth’s total biomass^1^. Per-cell catabolic rates and total biomass are presently unknown for microbial communities in the igneous aquifer beneath the sediment.

Because subseafloor communities reside at the interface between the biologically active surface world and the large geological reservoirs of biologically important chemicals, subseafloor microbial activities play a fundamental role in Earth’s biogeochemical cycles. This role is particularly visible in the organic-fueled activity of marine sedimentary life. Burial of reducing power (electron donors) in the form of organic matter and pyrite (FeS_2_) is the principal driver of Earth’s surface oxidation over geologic time^13^. Subseafloor life is the last biological filter through which organic matter passes on its way to burial and subduction. Among Earth’s surface and near-surface environments, marine sediment is the largest reservoir of carbon (including both the reduced carbon in organic matter and the oxidized carbon in sedimentary carbonates)^14^ and the third largest reservoir of nitrogen (after the atmosphere and continental crust)^15^.

**Fig. 1:**
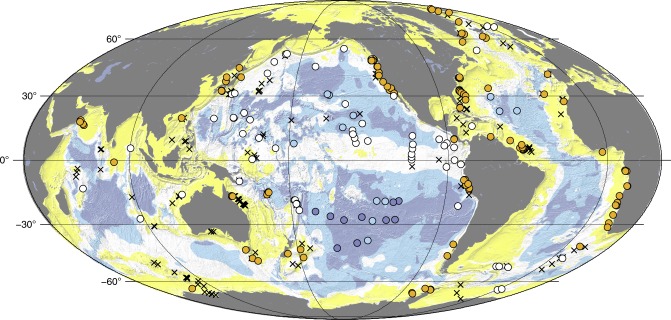
Global distributions of dissolved O_2_ and SO_4_^2−^ in subseafloor sediment. In yellow regions, dissolved SO_4_^2−^ is expected to disappear within the sediment and HCO_3_^−^ is expected to be the predominant net electron acceptor at greater depths. In white regions, dissolved SO_4_^2−^ is expected to penetrate throughout the sediment, from seafloor to igneous basement. Dark blue and light blue regions respectively represent the minimum and maximum areas over which dissolved O_2_ is expected to penetrate throughout the sediment from seafloor to basement^16^. SO_4_^2−^ reduction is expected to dominate net subseafloor organic oxidation in the yellow and white regions. O_2_ reduction is expected to dominate net subseafloor organic oxidation in the blue regions. Orange dots mark drill sites where SO_4_^2−^ disappears within the first 500 meters of sediment. White dots mark drill sites where SO_4_^2−^ penetrates the entire sediment sequence. Dark blue dots mark drill sites where dissolved O_2_ and dissolved SO_4_^2−^ penetrate the entire sediment sequence. Medium blue dots mark coring sites where O_2_ and SO_4_^2−^ penetrate to the bottom of the cores (meters to tens of meters) and may penetrate to basement. X symbols mark sites where the full extent of SO_4_^2−^ penetration is unknown, because SO_4_^2−^ is present in all measured samples, but the sedimentary sequence continues far below the last measurement. More information regarding the generation of this map is available in the Methods section

**Fig. 2:**
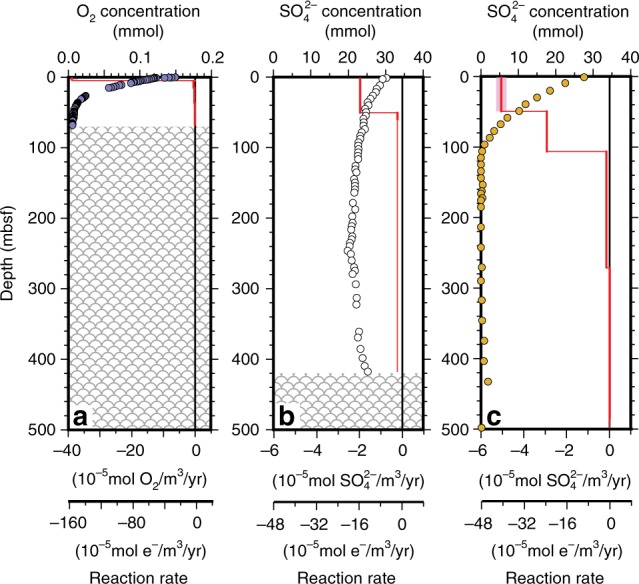
Profiles of net respiration rates at representative sites. **a** Profile of net respiration rate (O_2_ reduction rate) as a function of sediment depth at oxic IODP Site U1370 (dark blue zone in Fig. 1). **b** Profile of net respiration rate (SO_4_^2−^ reduction rate) as a function of sediment depth at anoxic ODP Site 1226, where dissolved SO_4_^2−^ is present throughout the sediment column (white zone in Fig. 1). **c** Profile of net respiration rate (SO_4_^2−^ reduction rate) as a function of sediment depth at anoxic ODP Site 984, where dissolved SO_4_^2−^ is depleted at a relatively shallow sediment depth (yellow zone in Fig. 1). Circles mark chemical concentration data color-coded to the Fig. 1 geographic zones in which the sites occur (dark blue, white and yellow, respectively). Red lines mark net respiration rates and pink bars mark uncertainty (first standard deviation). Crosshatched zones in A and B mark igneous basement

**Fig. 3:**
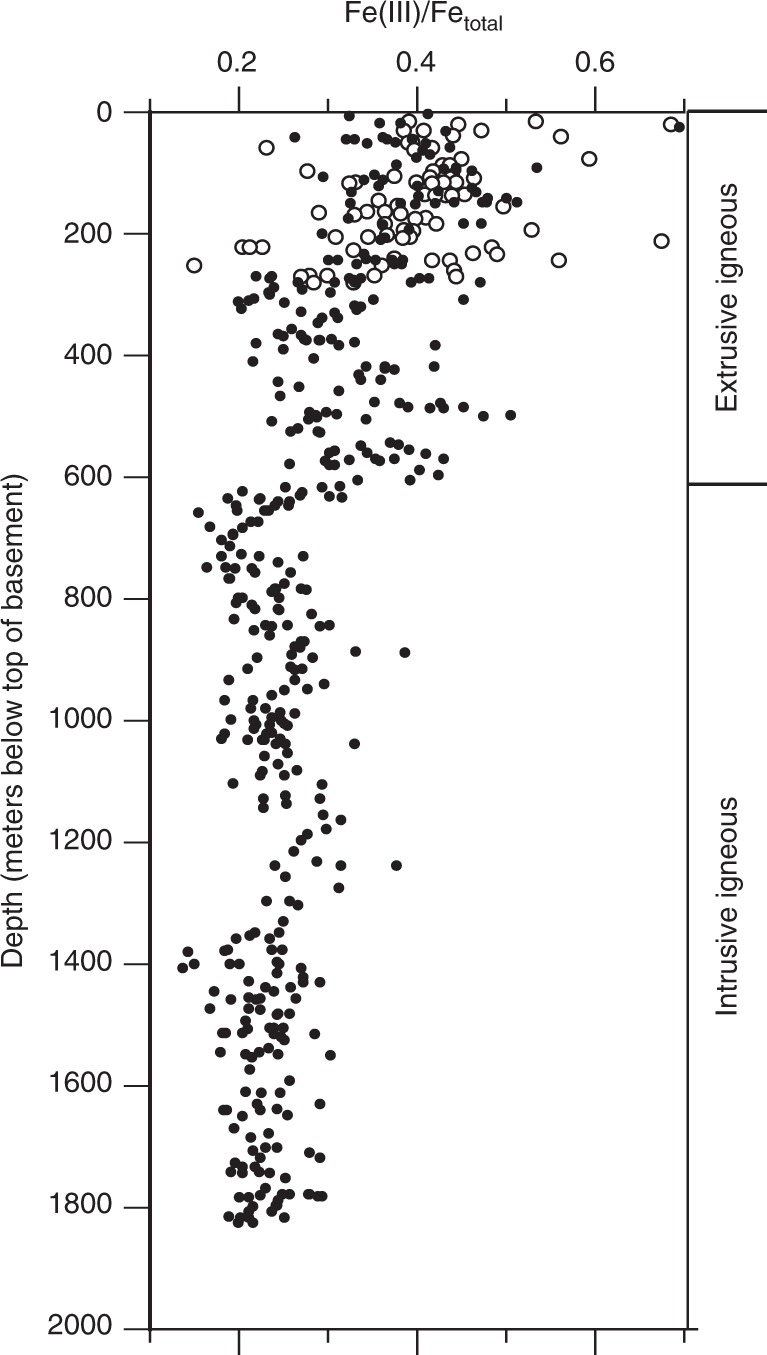
Profile of Fe oxidation ratio [Fe(III)/Fe_total_] as function of basement depth. Fe_total_ = Fe(III) + Fe(II). Higher ratios of Fe(III)/Fe_total_ show that a larger fraction of the total Fe is oxidized. Filled circles mark data from DSDP Hole 504B. Open circles mark data from ODP Hole 896 A. All data from Alt et al.^20^. Figure modified from Alt et al.^20^

The general geography of subseafloor catabolic activity is well constrained. Anaerobic activity dominates in the fast-accumulating sediment of continental margins and oceanic upwelling regions, where subseafloor respiration greatly outpaces the flux of dissolved oxygen (O_2_) into the sediment from the ocean. Aerobic activity dominates in the very slowly accumulating sediment of the deep open ocean, where subseafloor respiration rates are very low and the O_2_ flux keeps pace with respiration throughout the sediment column^16^ (Fig. 1). Rates of organic-fueled activity are highest in young, near-seafloor sediment and decline rapidly with increasing depth below seafloor^17^ (Fig. 2). Similarly, oxidation of igneous ocean crust is fastest for young crust (less than circa 10 Ma) and relatively slow thereafter^18^. Crustal oxidation is generally greatest where permeability is highest and seawater flow through the igneous aquifer is consequently fastest^19^. This is typically in discrete zones within the upper 200–500 m of the igneous sequence^20^ (Fig. 3).

Given the ubiquity of subseafloor life and its central role in chemical exchange between the surface and subsurface worlds, this review has three major objectives. The first objective is to synthesize present understanding of subseafloor microbes, their catabolic activities, and their global consequences. The second objective is to advance understanding of the factor(s) that ultimately limit rates of subseafloor activities. The third objective is to identify major gaps in scientific understanding of subseafloor communities and activities, and recommend approaches to filling those gaps.

### Diversities of subseafloor organisms and activities

Subseafloor sediment and the underlying basaltic aquifer are inhabited by many lineages of Bacteria^21–23^, Archaea^22,23^, and a subset of the Eukarya (fungi)^24,25^. Transcriptional data indicate that members of all three domains are active in subseafloor sediment^25^. Viruses are also abundant in both the sediment^26^ and the basaltic aquifer^27^.

Due to low per-cell energy fluxes^28^, selection is fierce in subseafloor sediment, leading both microbial diversity^29^ and cell concentration^1^ to decline exponentially with sediment depth and age. Broadly defined taxonomic groups (*Chloroflexi*, *Deltaproteobacteria*, *Firmicutes*) are present in relatively cool subseafloor sediment throughout much of the ocean^22,23,29–34^, suggesting that the consequences of this selection are relatively consistent at coarse taxonomic levels.

Geographic distributions of taxonomic richness and microbial abundance are largely unknown for the subseafloor igneous aquifer. However, different habitats within this environment are known to harbor distinctly different communities. For example, hot (65 °C) anoxic fluid in 3.5-Ma basalt of the Juan de Fuca Ridge (northeastern Pacific) contains abundant thermophilic anaerobes^3,35^, while relatively cold (3–4 °C) oxic fluid of the North Pond aquifer (central North Atlantic) is dominated by diverse *Proteobacteria*^36,37^. Communities from both aquifers are dominated by bacteria, with only trace concentrations of archaea^35,37^.

Subseafloor organisms glean energy from a broad range of redox reactions (e.g., disproportionation reactions, such as fermentation, and respiration reactions). In both the sediment and the igneous basement, electron acceptors (oxidants) for respiration include dissolved chemicals (O_2_, nitrate [NO_3_^−^], sulfate [SO_4_^2−^]) carried by diffusion or flow into the subseafloor, oxidized elements in minerals, and hydrogen peroxide (H_2_O_2_) from water radiolysis. In sediment, electron donors (reductants) include organic matter and reduced minerals deposited from the overlying world^28^, and hydrogen (H_2_) from in situ radiolysis of water^38^. In the igneous crust, electron donors include reduced elements in the rock (which originated in the underlying mantle)^18^, dissolved organic matter that enters with water from the overlying ocean^6^, and H_2_ from in situ water radiolysis^39^. Although in situ radiolysis may support subseafloor respiration in both the sediment and the underlying igneous aquifer, it may not have a net effect on subseafloor chemistry or global biogeochemical cycles, because it simultaneously creates reductant (H_2_) and oxidant (H_2_O_2_ and oxidized mineral species) in stoichiometric balance.

The traditional model of subsurface respiration assumes successive zones of reduction of O_2_, NO_3_^−^, oxidized manganese (Mn[IV]), oxidized iron (Fe[III]), SO_4_^2−^, and carbon dioxide (CO_2_) with increasing distance from the oxic surface world. In marine sediment, this sequence is vertical, with increasing depth below seafloor^40,41^ (Box 1). In aquifers with active fluid flow, the sequence depends on distance from the location where the fluid enters the aquifer from the surface^42^, e.g., the location where seawater enters the subseafloor igneous basement.

Most metabolic activities known to occur in subseafloor ecosystems were first discovered in surface or near-surface ecosystems. However, a few were first discovered in subseafloor sediment, including anaerobic oxidation of methane^43^, sulfate-reducing ammonium oxidation^44^, and microbial production of ethane and propane^45^.

The specific taxa responsible for individual metabolic activities are not yet known for most subseafloor environments^46^. The taxa responsible for specific activities may vary considerably in space and time; a recent metagenomic study of subseafloor planktonic communities in a cold oxic basalt aquifer found a high degree of metabolic functional redundancy over two years of sample collection, despite large shifts in community composition^37^.

### Global consequences of subseafloor biological activities

**Fig. 4:**
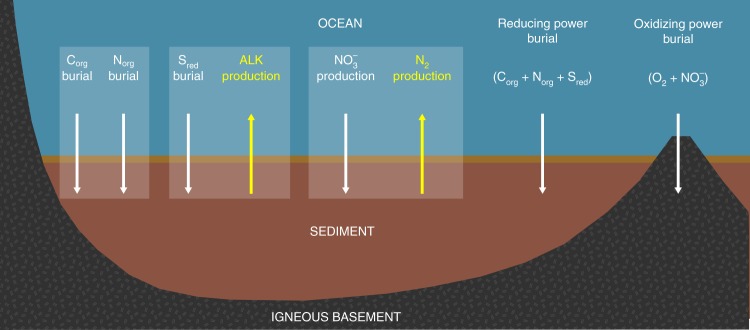
Major net chemical fluxes due to microbial activities in subseafloor environments. Subseafloor microbial activities control burial fluxes of organic carbon (C_org_), organic nitrogen (N_org_), and reduced sulfur (S_red_) (typically buried as iron sulfide), as well as production fluxes of alkalinity (ALK) and N_2_. In doing so, they control the rate at which reducing power is buried. Microbes in igneous basement contribute to burial of oxidizing power (via reduction of O_2_ and NO_3_^−^)

Subseafloor microbial activity has at least five major consequences for global biogeochemical cycles (Fig. 4). First, it plays an important role in the oxidation-reduction (redox) evolution of Earth’s surface and near-surface environments^47^. Burial of organic matter is the principal pathway for removing reduced material from the surface world and subsurface microbial activity limits the rate of organic burial. In other words, subsurface microbial activity is the final throttle on the primary driver of Earth’s surface oxidation. Second, pyrite (FeS_2_) produced as a result of microbial SO_4_^2−^ reduction in marine sediment is the principal sink for sulfur from the world ocean^48^. Third, this microbially driven pyrite production is a principal source of alkalinity to the world ocean^11^. These consequences of pyrite precipitation are significant for two reasons: sulfate and bicarbonate (the principal contributor to alkalinity) are the second and third most abundant anions in seawater (after chloride)^49^, and alkalinity influences the distribution of CO_2_ between the atmosphere and ocean by determining the speciation of dissolved inorganic carbon. Fourth, subseafloor NO_3_^−^ reduction decreases the amount of biologically fixed nitrogen available for marine biomass production (which is often N-limited)^50^. Fifth, subseafloor microbial activities play a significant role in creating and/or destroying resources of economic interest, including marine deposits of hydrocarbons^51^, phosphate^52^, dolomite^53,54^, and barite^55^.

### Impact of subseafloor activity on the global redox budget

Oxidation-reduction (redox) reactions transfer electrons from one chemical to another. This transfer constitutes one of the most significant processes on Earth’s surface. The primary driver of redox activity on Earth’s surface is oxygenic photosynthesis, which converts water and carbon dioxide into oxidized and reduced compounds (O_2_ and organic matter, respectively)^13^. Organic-fueled respiration reverses this process by oxidizing the organic matter.

Over Earth history, the ocean and atmosphere have been progressively oxidized by continuous burial of reduced biological products (principally organic matter and pyrite)^13^. This burial results from the failure of subsurface microbes to oxidize all of the organic matter within sediment and from the microbially mediated transfer of reducing power from organic matter to pyrite. Major manifestations of this long-term oxidation include the precipitation of banded iron oxide deposits during the Archean and early Paleoproterozoic^56^ and the appearance of abundant free O_2_ in the ocean and atmosphere^57^. Consequences of this long-term oxidation include Earth’s astonishing diversity of oxygen-reliant organisms^58^, metabolites^59^, and oxidized minerals^60^.

The rate of organic carbon production (net primary production) via photosynthesis is ~4.5 × 10^15^ moles C yr^−1^ in the ocean^61^ and ~4.7 × 10^15^ moles C yr^−1^ on land^62^. Organic carbon flux to the seafloor is ~2 × 10^14^ moles C yr^−1^ ^61,63^. Assuming a typical marine organic ratio of nitrogen to carbon in the seafloor organic flux (0.15)^64^, organic nitrogen flux to the seafloor is ~0.3 × 10^14^ moles N yr^−1^. Most of the organic-matter flux is ultimately oxidized, sustaining life at the seafloor and within the sediment. However, a significant fraction of the organic flux to the seafloor is permanently buried, rather than respired; the net organic C burial rate in marine sediment is in the range of 0.2 × 10^14^ moles C yr^−1^ ^61^ to 0.7 × 10^14^ moles C yr^−1^ ^63^ (Table 1). Most of the flux to the seafloor (70–85%) and the long-term burial (~90%) occurs in coast and shelf environments, in water depths shallower than 200 meters^61,63^.

**Table 1:** Major net chemical fluxes due to microbial activities in subseafloor environments. Organic carbon burial flux estimates from Sarmiento and Gruber^35^ and Dunne et al.^37^. Estimates of NO_3_^−^ flux to sediment and N_2_ flux from sediment from Gruber^50^. All other fluxes calculated as described in the text. As described in the text, the extent to which microbial activities are responsible for the net oxidizing flux into the igneous basement is not yet known

Process	Net flux into sediment	Net flux out of sediment	Net flux into igneous basement
Organic C burial	1.6–6.5 ×10^13^ mol C yr^−1^		
Organic N burial	2.4–9.8 × 10^12^ mol N yr^−1^		
Reduced S burial (FeS_2_ precipitation)	2–9 × 10^12^ mol S yr^−1^		
Reducing power burial (C_org_ + N_org_ + S_red_)	1–4 × 10^14^ mol electron equivalents yr^−1^		
Oxidizing power burial (O_2_ & NO_3_^−^ reduction)			2.5 ± 1.3 × 10^12^ mol electron equivalents yr^−1^?
Alkalinity production from FeS_2_ precipitation		4–18 × 10^12^ mol alkalinity equivalents yr^−1^	
NO_3_^−^ reduction	3.6 ± 1.2 × 10^11^ mol N yr^−1^		
N_2_ production		3.6 ± 1.2 × 10^11^ mol N yr^−1^	

By oxidizing organic matter in marine sediment, microbial communities reduce the mean oxidation state of the ocean and atmosphere. From a global redox perspective, this effect is a subseafloor manifestation of the same processes that consume organic matter in the surface world. Net microbial respiration in marine sediment generally consumes both oxidants (electron acceptors) and reductants (electron donors) that originated in the surface photosynthetic world. The principal net oxidants include dissolved species (O_2_, NO_3_^−^, SO_4_^2−^) that diffuse into sediment from the overlying ocean, and solid-phase oxidized metals (e.g., Fe[III] and Mn[IV] in minerals). The principal net reductant is buried organic matter. The principal products of organic-fueled respiration in marine sediment are dissolved inorganic carbon (DIC = HCO_3_^−^ + CO_3_^2−^ + dissolved CO_2_) and dissolved nitrogen species. This DIC generally has one of three fates: some returns to the ocean via diffusion and/or advection as DIC, some is incorporated into carbonate minerals (e.g., CaCO_3_) and remains buried, and some is buried as DIC where sediment accumulates faster than chemicals can diffusively return to the ocean. Nitrogen in buried organic matter is released by organic degradation as ammonium (NH_4_^+^) and amines^50^. Much of this reduced nitrogen is oxidized to NO_3_^−^ and returns to the ocean. Some of this NO_3_^−^ is transformed to N_2_ (denitrified) during its migration to the ocean. Some of the reduced nitrogen is buried as dissolved NH_4_^+^, incorporated into mineral phases, or transformed to N_2_ by anaerobic processes^44^.

For this review, we quantify sedimentary redox fluxes in terms of the number of electrons transferred during reduction from the chemical species stable in contact with Earth’s oxygenated atmosphere (e.g., CO_2_, SO_4_^2−^, NO_3_^−^) to the reduced chemical species that persist in subseafloor sediment (e.g., organic matter, FeS_2_) (Methods). We similarly quantify basement redox fluxes in terms of the electron numbers required to oxidize the reduced chemicals in basalt. Previous studies have presented redox fluxes in terms of O_2_ equivalents^47^ or H_2_ equivalents;^13^ one O_2_ equivalent corresponds to four electron equivalents and one H_2_ equivalent corresponds to two electron equivalents.

The total electron-equivalent burial rate in marine sediment, including reduced C, N and S, is 1 × 10^14^ to 4 × 10^14^ moles e^−^ yr^−1^ (Table 1). The marine sedimentary organic carbon burial rate of 1.6 × 10^13^ to 6.5 × 10^13^ moles C yr^−1^ ^61,63^ equals 6.4 × 10^13^ to 2.6 × 10^14^ moles e^−^ yr^−1^. Assuming an N/C ratio equal to typical marine organic matter (0.15)^64^, the marine sedimentary organic nitrogen burial rate is 2.4 × 10^12^ to 9.8 × 10^12^ moles N yr^−1^ (Table 1). Electron-equivalent burial as organic nitrogen (1.9 × 10^13^ to 7.8 × 10^13^ moles e^−^ yr^−1^) is ~1/3 the rate of electron-equivalent burial as organic carbon.

Marine sedimentary communities also modulate redox pathways by transferring electrons from one buried chemical species to another, most notably by transferring electrons from organic matter to iron and sulfur, leading to FeS_2_ formation. In this case, reducing equivalents (electron equivalents) that were originally buried with organic matter continue to be buried with the sulfur and iron to which they were transferred. This transfer of electrons is significant. Given an average burial ratio of organic carbon to reduced sulfur in marine sediment (7.5 mole C/mole S)^65^ and the organic burial rates mentioned above, the rate of reduced sulfur burial, principally as FeS_2_, is 2 × 10^12^ to 9 × 10^12^ moles S yr^−1^ (Table 1). The burial rate of electron equivalents as reduced sulfur (1.4 × 10^13^ to 6.3 × 10^13^ moles e^−^ yr^−1^) is approximately 1/4 the rate of electron-equivalent burial as organic carbon and slightly lower than the rate of electron-equivalent burial as organic nitrogen.

Oxidation of the fractured igneous basement that underlies the sediment also plays a role in global redox cycling, by reducing the oxidation state of the ocean and atmosphere^47^. As seawater hydrothermally circulates through this aquifer, electron acceptors from the ocean, principally O_2_ and NO_3_^−^, oxidize reduced elements, principally iron and sulfur, in igneous minerals^18^. This circulation introduces electron acceptors to the upper crust throughout much of the ocean; dissolved O_2_ and NO_3_^−^ is present in Eocene and Miocene basalt of the eastern Pacific^21^, Pleistocene to Cretaceous basalt of the central South Pacific^16^, and Miocene basalt of the North Atlantic^66^. The resulting oxidation of 1.7 ± 1.2 ×  10^12^ moles Fe yr^−1^ and 1.1 ± 0.7 × 10^11^ moles S yr^−1^ ^18^ reduces the ocean and atmosphere by 2.5 ± 1.3 × 10^12^ moles e^−^yr^−1^ (Table 1). The extent to which microbial respiration drives this oxidation is unknown, because dissolved O_2_ and NO_3_^−^ abiologically oxidize reduced iron and sulfur^67^.

Multiple lines of evidence suggest that microbes harvest at least some of the energy from the mineral-oxidation reactions in the igneous basement. Microbial communities are routinely sampled from the basement aquifer^3,36^, members of these communities readily colonize fresh mineral surfaces^68,69^, and chemical data indicate that microbes play a role in late stages of rock alteration within the aquifer (e.g., SO_4_^2−^ reduction, methanogenesis)^4,5^. However, the extent to which subseafloor communities utilize the energy from these reactions is unknown. More importantly for this review, the net effect of that harvest on the global redox cycle is also unknown. For example, it is not yet clear if microbial communities enhance the total rate of basement oxidation by increasing the extent to which these reactions penetrate into the rock.

### Impact on the global sulfur cycle

As the primary sink of sulfur from the ocean^48^ and a major source of alkalinity to the ocean^11^, SO_4_^2−^ reduction coupled to FeS_2_ precipitation in marine sediment directly affects ocean chemistry and indirectly affects atmospheric chemistry and climate. Sulfate is the dominant electron acceptor in the anoxic sediment that blankets ~60% of the seafloor. However, only a fraction of the sulfur reduced globally in marine sediment is precipitated as FeS_2_. Sulfur is reduced at the shallowest depths and highest rates in coastal environments, where up to 95% of the resulting sulfide diffuses upward and is re-oxidized^70^. In open-ocean environments, such as the equatorial Pacific, SO_4_^2−^ is reduced at greater sediment depths and iron concentration is great enough that ~100% of the reduced sulfur precipitates as FeS_2_^71^.

The natural (pre-anthropogenic) flux of sulfur to the ocean is almost entirely via rivers. A recent estimate of this flux is ~2 × 10^12^ moles S yr^−1^ ^48^. This estimate ignores rapid cycling of sulfur between the ocean and the immediately overlying atmosphere. It also ignores rapid microbial cycling of sulfur reduction and re-oxidation in shallow marine sediment and the water column of oxygen-minimum zones. Again ignoring these rapid cycles, the flux of sulfur from the ocean is almost entirely via metal sulfide precipitation (primarily FeS_2_ in marine sediment)^13,48^. As discussed above, we estimate the rate of reduced sulfur burial in marine sediment to be 2 × 10^12^ to 9 × 10^12^ moles S yr^−1^ (Table 1).

### Impact on alkalinity and atmospheric CO_2_

Precipitation of metal sulfide following SO_4_^2−^ reduction removes the anionic charge of SO_4_^2−^ from the ocean. This charge is balanced by production of alkalinity^11^ (alkalinity is equal to the sum of positive charge equivalents minus the equivalents of negative charge from the conjugate bases of strong acids [Cl^−^ and SO_4_^2−^])^49^. Consequently, precipitation in marine sediment of 2 × 10^12^ to 9 × 10^12^ moles reduced S yr^−1^ generates 4 × 10^12^ to 1.8 × 10^13^ moles of alkalinity equivalent yr^−1^ (Table 1). Given these estimates and a riverine primary alkalinity flux to the ocean of 1.3 × 10^13^ mole equivalents yr^−1^ ^72^, microbially induced precipitation of sedimentary FeS_2_ generates 24–58% of the annual primary alkalinity flux to the ocean. CO_2_ partial pressure (*p*_CO*2*_) is highly sensitive to the value of alkalinity; for present-day ocean chemistry, a 25% increase in alkalinity will decrease atmospheric *p*_CO*2*_ by more than an order of magnitude (Methods).

### Impact on the global nitrogen cycle

Ongoing burial of organic nitrogen is the primary sink of nitrogen from the atmosphere and ocean. Furthermore, microbial NO_3_^−^ reduction (denitrification) linked to organic matter oxidation in marine sediment is the principal sink of fixed nitrogen from the ocean; the rate of sedimentary NO_3_^−^ reduction is about a factor of two greater than the rate of denitrification in the water column and an order of magnitude larger than the rate of organic nitrogen burial^50^. In the absence of sedimentary denitrification, most marine ecosystems would not be nitrogen-limited. Most of this denitrification occurs in coast and shelf sediment, where the flux of organic matter to the seafloor is highest.

### Impact on geological resources

Subseafloor microbial activities also play significant roles in creating and destroying resources of economic interest. The importance of subseafloor microbial activities is most fully delineated for destruction of subseafloor hydrocarbon resources^51^. More than 90% of the methane produced in marine sediment is consumed by anaerobic communities within the sediment^73^. Biodegradation is also extensive in relatively cool (50 °C) marine oil reservoirs, which have typically lost up to ~50% of their mass of C_6+_ hydrocarbons^51^. Biodegradation decreases greatly as a function of reservoir temperature. Evidence of oil biodegradation is generally absent from reservoirs warmer than 80 °C, suggesting that the thermal limit to life in the reservoirs is close to that temperature^51^. Although microbial roles are less thoroughly studied for production and destruction of other economic resources, rates and subsurface locations of organic oxidation, sulfate reduction and methane production are known to play central roles in precipitating sedimentary deposits of phosphate^52^, barite^55^, dolomite^53,54^, and gas hydrates. Microbial activities may also play central roles in formation of marine metal deposits, including hydrothermal sulfides^74^.

### The limits of subseafloor activity

Since active subseafloor communities are present throughout the world ocean, a paramount biogeochemical mystery has been the failure of those communities to completely consume the electron donors present in subseafloor environments. Even in oxic marine sediment, traces of organic matter may survive for 100 Myrs or more^16^. Reduced iron and reduced sulfur persist in subseafloor basalt for more than 100 Myrs^18^. This failure plays a significant role in the redox evolution of Earth’s atmosphere and ocean, because, as described above, sequestration of organic matter in marine sediment is a principal sink for reducing power from the surface world. Furthermore, failure of microbial communities to oxidize all of the iron and sulfur in the subseafloor igneous aquifer leaves oxidizing power in the ocean that might have otherwise been lost to mineral oxidation. The failure to consume all of the buried organic matter also decreases the influence of subseafloor respiration on global cycles of sulfur, nitrogen and alkalinity. The persistence of organic matter in marine sediment is also crucial for thermogenic formation of major hydrocarbon resources, because oil and gas cannot form in the absence of their feedstock.

### Not all sedimentary organic matter is consumed

Organic oxidation rate is generally highest at the seafloor and decreases with sediment depth, declining rapidly near the seafloor and increasingly slowly with greater distance from the seafloor. This relationship is typically modeled as a power-law function^17,75,76^. The basic reaction series can be simplified as follows:

1 $${\mathrm{6CH}}_{\mathrm{2}}{\mathrm{O}}_{{\mathrm{POM}}}--- > \left( {{\mathrm{CH}}_{\mathrm{2}}{\mathrm{O}}} \right)_{{\mathrm{6}}\;{\mathrm{(dissolved)}}}--- > 2\left( {{\mathrm{CH}}_{\mathrm{2}}{\mathrm{O}}} \right)_3 \\ \hskip 10pt+\,{\mathrm{3SO}}_{\mathrm{4}}^{2 - }--- > {\mathrm{6CO}}_2 + {\mathrm{3H}}_{\mathrm{2}}{\mathrm{S}}$$

Standard models of organic persistence in marine sediment assume that the first step is the rate-limiting step^75^. However, if organisms are operating at their minimum harvestable reaction affinity^77^, catabolic reactions cannot proceed faster than reactants appear or reaction products disappear at the reaction location^78^. In this case, the rate-limiting step may occur later in the reaction series, such as the final respiration step of the above sequence, which will be limited by the rates at which SO_4_^2−^ is introduced and the rate(s) at which CO_2_ and H_2_S are removed (Box 2).

This dependence of organic oxidation rate on chemical fluxes and minimum reaction affinity will naturally lead to the typical depth-dependence of organic oxidation rate (Box 2). Because the time required for chemical diffusion is proportional to the square of diffusive distance^79^, where advection is insignificant (below the depth of bio-irrigation), organic oxidation rate must decline exponentially with distance from sources of catabolic reactants and sinks of catabolic products (Box 2). Where electron acceptors and catabolic products respectively diffuse from and to the ocean above, the rate of organic oxidation will decline exponentially with depth beneath the seafloor.

Most quantitative models of the decline in bulk organic oxidation rate with sediment depth assume that organic persistence results from organic molecular structure, with selective preservation of the least reactive (most carbon-rich) compounds^75,76^. These models are variations on Berner’s 1964 G Model, which assumed organic oxidation rate to be proportional to organic matter concentration^76^. More recent models assume that bulk organic oxidation rate declines with sediment depth and age because increasingly recalcitrant pools of organic matter are successively depleted^17^. This assumption is consistent with laboratory experiments that show sequential utilization of different sedimentary organic carbon pools by aerobic heterotrophs^80^. Despite this consistency, reliance on relative degradability to explain organic oxidation rates is problematic for the following reasons. First, the molecular composition of these organic pools is never identified by studies that assume them to model organic oxidation rates^17,76^. Second, the degradability of an organic-matter pool, such as kerogen (the fraction of organic matter insoluble to organic solvents) in deeply buried sediment, is not inherent to the organic molecules that compose that pool, but depends on the environment in which the organic pool is present^81^. For example, 365-million-year-old organic matter is quickly degraded by microbes when exposed by erosion^82^. Third, preferential degradation or persistence of different classes of organic compounds may be better considered as a function of community metabolic capabilities than as an intrinsic property of organic molecules^83^. Fourth, preferential persistence of the most carbon-rich compounds is difficult to reconcile with preservation of relatively low C/N ratios for tens of Myrs^84,85^, and with subseafloor respiration of C/N ratios close to the average marine (Redfield) value for tens of Myrs^16,29,86^.

Other studies have proposed that organic matter persists because it is adsorbed to mineral grains^85,87,88^. This hypothesis is consistent with a generally close association between high bulk organic concentrations and abundance of fine-grained minerals^65^. However, reliance on organic adsorption to minerals as the primary explanation of organic persistence in marine sediment is difficult to sustain for the following reasons. First, it is difficult to reconcile the molecular integrity of some compounds, such as certain fatty acids and pigments, with the solubilization that must precede adsorption^89^. Second, discontinuous distribution of organic matter renders binding to mineral grains an insufficient explanation of organic persistence in continental-margin sediment^65,90^. Third, much of the adsorption to minerals is easily reversed and ultimately cannot preserve organic matter^91^.

These different explanations have fundamentally different implications for the relationship between organic oxidation rates and organic-matter persistence in deep old sediment. To the extent that organic oxidation rate depends on minimum reaction affinity and diffusion rates of dissolved catabolic reactants and products (Box 2), organic matter can persist simply because oxidation rate declines with sediment depth. In contrast, selective-preservation hypotheses assume that organic oxidation rate declines because residual organic molecules are chemically recalcitrant. Finally, the mineral adsorption hypothesis could be used to predict that organic oxidation rate declines because residual organic matter is strongly adsorbed to minerals.

Demonstration of increased organic recalcitrance or increased mineral adsorption with sediment age is necessary to respectively support the hypotheses that organic recalcitrance or mineral adsorption drives the decrease in organic oxidation rate with increasing sediment depth and age. However, neither test is sufficient to prove either hypothesis, because organic recalcitrance and/or mineral adsorption might increase with sediment age even if they do not drive the decrease in organic oxidation rate with sediment depth and age.

Persistence of electron donors in subseafloor sediment is not limited to solid-phase organic matter. For example, the turnover times of dissolved fatty acids in sulfate-reducing subseafloor sedimentary ecosystems ranges from approximately tens to hundreds of years (Methods). These relatively long turnover times of dissolved electron donors are not explainable by chemical recalcitrance or adsorption to minerals. However, they are a natural consequence of microbial thermodynamics coupled to subseafloor timescales of chemical diffusion (Box 2).

The causal relationship between organic oxidation rates and organic-matter persistence may differ from one marine sedimentary environment to another. Diffusion of chemical species may limit organic oxidation rates in fast-accumulating anoxic sediment, where organic oxidation reactions operate at minimum harvestable affinities of reaction. However, in sediment where electron acceptors are abundant and reaction products are scarce, organic persistence may result from mineral shielding or molecular inaccessibility of the organic matter. Such circumstances might apply in the slowly accumulating organic-poor oxic sediment that blankets much of the abyssal ocean^16,85^.

### The igneous subseafloor is not fully oxidized

The factors that limit oxidation rates in the igneous subseafloor are rarely addressed explicitly. The permeability of un-fractured basalt is extremely low and rock alteration is closely associated with fractured surfaces^92^. Given these points, persistence of reduced iron and sulfur in subseafloor basalt can most simply be ascribed to physical inaccessibility of the reduced elements within the un-fractured rock. This ascription is a variation on the diffusive-timescale explanation that we present for sedimentary organic oxidation above and in Box 2; however, in the igneous basement, permeability is so low that chemical diffusion of electron acceptors into un-fractured rock is extremely slow, even for sub-millimeter distances.

The persistence of dissolved organic matter (DOM) in the igneous aquifer is more enigmatic. DOM enters the aquifer with water from the overlying ocean^6^. Because DOM is in the water, not the rock, it is in contact with dissolved electron acceptors. Although some DOM is consumed within the aquifer, DOM is present along the entire flow path. In the cool oxygenated aquifer of North Pond (North Atlantic), DOM has radiocarbon model ages of several thousand years^6^. These model ages are significantly older than the residence time of water in the aquifer or the residence time of deep water in the North Atlantic, suggesting ongoing supply of aged organic matter to the deep ocean and the aquifer.

### Microbial activities and the subseafloor limit to life

The preceding paragraphs and Box 2 focus on limits to subseafloor activity in environments where organisms are present and metabolically active. In subseafloor environments, fluxes of bioavailable energy may be too low to maintain community function under physical conditions that differ notably from physical limits to life in other environments. For example, the high-temperature limit to oil and gas bioalteration (80–90 °C)^51^ is considerably lower than the high-temperature limit to life in energy-rich and nutrient-rich autoclave conditions (122 °C)^8^. This difference illustrates that the high-temperature limit to life may vary from one environment to another as a function of bioavailable energy flux or nutrients^9,51^.

Factors other than temperature may also sterilize subseafloor systems. More precisely, changes in chemical conditions might sterilize subseafloor environments after multi-Myr intervals of selection for previous chemical conditions. For example, the oxic condition of abyssal clay sequences for tens of Myrs may drive obligate anaerobes in the clay to extinction, to be followed by local extinction of aerobes if sedimentation rates increase and dissolved O_2_ disappears from the clay.

### Future directions

As indicated in the preceding sections, many key issues remain to be resolved. Global rates of subseafloor microbial activities and their consequences are only roughly quantified. For example, recent estimates of organic carbon burial in marine sediment differ by ~4 × ^61,63^ and recent estimates of global SO_4_^2−^ reduction rate in marine sediment differ by ~7.5× ^93,94^. Our estimates of FeS_2_ precipitation rate, alkalinity production rate and organic nitrogen burial rate in marine sediment are based on estimates of organic carbon burial rate and also vary by ~4×. This lack of precision is exacerbated by incomplete understanding of microbial processes in the subseafloor. For example, the extent to which microbes participate in redox alteration of inorganic phases in subseafloor basalt and sediment remains to be determined. More precisely, the extent to which microbes harvest the energies of mineral-altering redox processes that can proceed abiologically is not known. Furthermore, the extent to which microbes actively enhance mineral alteration, e.g., by mining more deeply or more rapidly into minerals and/or rock than done by abiotic alteration, is also unknown.

The factors that limit rates of subseafloor catabolic activities are crucial to the operation of Earth’s biogeochemical cycles but not fully understood. Consideration of reaction affinities and dissolved chemical transport suggests that diffusion rates of catabolic reactants and products play an important and previously under-recognized role in limiting subseafloor catabolic rates and, thereby, sustaining the persistence of electron donors in subseafloor environments.

The specific taxa responsible for individual activities are not known for most subseafloor environments^46^. The metabolic networks that sustain their communities for millions of years are largely unexplored. The nature and extent of communication between cells in subseafloor communities is unexamined. At the most fundamental biological level, the genomic properties and patterns of gene expression that enable lineages of subseafloor microbes to survive at extraordinarily low rates of activity for millions of years remain unknown^28^.

Better quantification of subseafloor microbial activity rates and their consequences will require integration and quantitative analysis of data from diverse studies. For example, more accurately quantifying global burial rates of organic carbon, organic nitrogen and sulfide in marine sediment will require synthesis of geochemical data from shallow and deep sediment of coastal environments, continental shelves and the open ocean. And precise quantification of sulfide burial’s effect on ocean alkalinity and atmospheric chemistry will require quantification of both sulfide burial and sulfate flux to the ocean.

Deeper understanding of subseafloor activities and their consequences will require Earth and life scientists to deliberately integrate techniques from diverse fields (e.g., molecular biology, aqueous geochemistry, sedimentology, petrology and mineralogy) to test key hypotheses. For example, fuller understanding of how dissolved chemical transport and reaction affinities limit rates of subseafloor activities – and ultimately control global biogeochemical fluxes – will require integrated study of in situ abilities to undertake specific catabolic reactions, in situ reaction rates, and in situ energies of reaction.

Hypothesis-driven laboratory experiments and direct study of natural communities will both be crucial to advance understanding of the physiological features, metabolic strategies, and genomic properties that enable lineages of subseafloor microbes to persist at extraordinarily low rates of activity for millions of years. Laboratory experiments are necessary to elucidate the features and strategies that enable microbes to thrive or survive under specific subseafloor conditions, at least on laboratory timescales, and the genes that code for those features and strategies. Direct studies of natural subseafloor communities are needed to delineate the conditions in which they live, to characterize microbial interactions in subseafloor communities, and to identify features and strategies that enable microbial lineages and communities to thrive or survive under subseafloor conditions for many millions of years.

With both laboratory experiments and natural-sample studies, a broad range of analytical approaches is crucial to understand the organisms and communities. Genomic studies are necessary to know what capabilities are available. Transcriptomic studies are needed to understand which capabilities are expressed. Biogeochemical studies are required to determine in situ reaction rates, in situ energies of reaction, and consequences of microbial activities. Exacting studies of organic composition and mineral composition are necessary to identify the substrates available to subseafloor organisms and the consequences of microbial activities. Natural-community studies often benefit from explicitly placing them in a temporal context, e.g., by repeatedly sampling a subseafloor flow path over time^37^, by sampling crust of very different ages, or by treating data from younger samples in the same sediment column as potentially representative of earlier stages in the history of a subseafloor community^29,95,96^ or habitat. Such temporal context is crucial for understanding how subseafloor communities are assembled. It is also critical for understanding how their biogeochemical roles and consequences vary throughout the subseafloor in space and time.

Individual studies need not integrate across many categories of study. Appropriate laboratory experiments can bring great value whether they are tied to specific field sampling programs or not. Similarly, well-posed natural-sample studies can effectively address key problems (e.g., quantification of specific biogeochemical fluxes or identification of globally consistent genomic and/or transcriptomic patterns) without close integration to other categories of natural-sample study or to laboratory experiments. Ultimately, however, each category of study derives greater strength by integration with one or more of the other categories. For example, laboratory studies inform the understanding of data from subseafloor samples, and subseafloor data inform the hypotheses that drive the laboratory experiments. Understanding of natural subseafloor communities and their consequences also benefits from synthesizing results of different approaches to natural samples. For example, biogeochemical results might explain why a specific capability that is present is not expressed, and genomic studies might explain why a reaction that is energetically feasible does not occur.

As shown by these examples, close integration of biological, chemical and physical approaches will ultimately be necessary to further advance understanding of subseafloor life and its biogeochemical consequences. This integration will require both Earth and life scientists to begin thinking of many geological and geochemical features (such as organic recalcitrance and rates of subseafloor redox processes) as ecosystem properties, rather than a priori environmental features.

Box 1 Co-occurrence of terminal electron-accepting processes The standard model of redox zonation in marine sediment assumes successive zones of reduction of O_2_, NO_3_^−^, oxidized manganese [Mn(IV)], oxidized iron [Fe(III)], SO_4_^2−^ and CO_2_ with increasing sediment depth. These chemicals, and the material they oxidize, enter sediment at the seafloor; solid phases (manganese- and iron-bearing minerals, organic matter) are deposited with sediment, O_2_, NO_3_^−^ and SO_4_^2−^ enter in dissolved form, and most dissolved CO_2_ is generated in the sediment by organic oxidation.

This succession of redox zones is generally believed to result from thermodynamic competition, in which organisms that use the actively reduced electron acceptor (e.g., O_2_) drive electron-donor concentrations too low to be energetically exploited in combination with less competitive electron acceptors (e.g., CO_2_)^114^. The successive redox activities have been described as mutually exclusive, with less competitive electron acceptors unused until the most competitive electron acceptor is exhausted^41^. For example, it has been assumed that SO_4_^2−^ is not reduced until accessible Fe(III) is exhausted^114^.

This standard model explains many broad patterns of subseafloor metabolism. It explains the near-seafloor succession of net oxidant consumption in anoxic sediment throughout much of the ocean^40,41^. It explains the dominance of oxic respiration over almost 40% of the seafloor (Fig. 1), where sediment accumulates very slowly and dissolved O_2_ penetrates the sediment to basement^16^. It explains reversed sequences of net redox activities where oxic seawater diffuses upward into anoxic sediment from the underlying basaltic aquifer^16,21,66^ and where sulfate-bearing water diffuses upward into sulfate-depleted sediment^21,115^.

Some patterns of subseafloor respiration modify the standard zonation, but do not necessarily challenge the assumption that the different processes are mutually exclusive. First, discrete zones of net Fe(III) reduction and net Mn(IV) reduction occur deep beneath zones of net SO_4_^2−^ reduction in some sediment^21^. These occurrences can be explained by original deposition of much higher oxidized metal concentrations or much lower organic matter concentration in deep metal-reducing zones than in shallower deposits. Second, isotopic data, transcriptomic data and radiotracer data have respectively been interpreted as evidence of NO_2_^−^ oxidation^116^, NO_3_^−^ reduction^25^ and SO_4_^2−^ reduction^117^ in sediment deep beneath the last occurrences of measurable O_2_, NO_3_^−^ and SO_4_^2−^. These processes can only occur at these depths if cryptic processes, such as reduction of H_2_O_2_ from water radiolysis^38^, sustain in situ production of the necessary electron acceptors (O_2_, NO_3_^−^, SO_4_^2−^).

A third pattern is consistent with thermodynamic competition, but disproves the assumption that the competing processes are mutually exclusive. As indicated by the dashed arrows in this figure, these processes often co-occur. O_2_ reduction and NO_3_^−^ reduction co-occur at low dissolved O_2_ concentrations^65^. NO_3_^−^ reduction and Mn(IV) reduction also often co-occur^65^. SO_4_^2−^ reduction and methanogenesis commonly co-occur in marine sediment^11,28,45^, along with Fe(III) reduction^28^, throughout sequences that span hundreds of meters and were deposited over millions of years. Fe(III) reduction has also been observed in the methanogenic zone^42^ and the sulfate-reducing zone of continental aquifers^118^.

Studies of SO_4_^2−^ reduction, Fe(III) reduction and methanogenesis indicate that where these processes co-occur, they may compete for electron donors but operate at the same in situ energy of reaction^28^. This in situ energy closely matches the minimum reaction affinity, *A* (*A* *=* *−ΔG* of the reaction)^119^, believed necessary for an organism to sustain an energy-conserving reaction^77,108,109^. Estimates of minimum affinities of energy-conserving anaerobic reactions are generally in the range of 2–5 kJ per mole of electrons transported^77,109^. Affinities in this range have been observed for multiple reactions in both shallow^44,77^ and deep^28,44^ marine sedimentary ecosystems.

Co-occurrence of these competing reactions may aid the organisms that rely on them, by removing reaction products and/or creating reactants. Such removal and/or creation may amplify rates of community activity by returning affinities of multiple reactions to the minimum values required to harvest the reaction energy. For example, co-precipitation of dissolved Fe(II) and dissolved sulfide increases affinities of both Fe(III) reduction and SO_4_^2−^ reduction^28^. Oxidation of methane (CH_4_) by SO_4_^2−^ reduction or Fe(III) reduction increases the affinity of methanogenic reactions, enabling methanogens to generate more CH_4_ that can in turn fuel the sulfate-reducing and Fe(III)-reducing reactions^28,109,118^.

In summary, as each successive electron-accepting process is initiated, it may hold electron donor concentrations too low to be energetically exploited in combination with other electron acceptors^114^. However, this competitive exclusion holds only until the competing reactions yield minimum harvestable affinities of reaction. At least for Fe(III) reduction, SO_4_^2−^ reduction and methanogenesis, concentrations of reactants (including electron donors) and products yield equivalent affinities of reaction and sustain mutually competing processes deep into subseafloor sediment^28^.

Box 2 Dependence of organic oxidation rate on diffusion timescale If the affinity of a catabolic reaction drops below the minimum value necessary to sustain biological energy conservation (Box 1), the energy of reaction is not biologically harvestable until the affinity of the reaction rises again. In short, the rate of catabolic activity is limited by the flux of bioavailable energy^78^. As the reaction proceeds, its affinity decreases. When it drops below the minimum value, energy is no longer conserved and the reaction stops until more reactant is introduced or reaction product is removed.

This relationship between the affinity of a reaction and the rate of that reaction is exemplified by chemostats, in which growth rate depends on the rate that reactants are added and products are removed. However, in a chemostat, reactants (e.g., O_2_, NO_3_^−^, SO_4_^2−^) are typically added by fluid inflow and reaction products [e.g., DIC] are removed by fluid outflow. In contrast, in most marine sediment, catabolic reactants are added and catabolic products are removed via diffusion. Because the timescale of diffusion is proportional to diffusive distance squared^79^, the rates of reactant introduction and product removal via diffusion decrease strongly with distance to the reactant source and distance to the product sink. In effect, increasing sediment thickness drives subseafloor ecosystems toward being closed systems^40^.

In other words, the rate of catabolic activity is expected to decrease with distance from where a reactant is introduced or a product is lost, due to the decreasing rate of diffusive transport, even if the lability and concentration of organic matter are constant throughout the sediment column (Methods). The effect of diffusive distance is small for reactants produced in situ^38^, such as radiolytic H_2_, and products removed in situ, such as sulfide precipitated in FeS_2_ at the location of sulfide production. However, this effect can be significant for reactants and products that diffuse to or from elsewhere in the sediment column (e.g., for O_2_, NO_3_^−^ or SO_4_^2−^ that diffuses into the sediment from the overlying ocean, and for DIC that diffuses from the sediment into the ocean).

We illustrate this effect with DIC production by sulfate-reducing oxidation of a carbohydrate (CH_2_O + 0.5 SO_4_^2−^ = > HCO_3_^−^ + 0.5 HS). We focus on HCO_3_^−^ because DIC is predominantly HCO_3_^−^ in marine sediment. For simplicity, this example assumes that concentrations of HS^−^, CH_2_O and SO_4_^2−^ are constant or proportionally change to a much lesser extent than HCO_3_^−^. Given this assumption and reaction affinity at the minimum biologically harvestable value, reaction rate directly depends on the net rate of DIC diffusion at each depth. Expansion of this model to include catabolic reactants, additional reaction products and/or additional reactions (e.g., organic solubilization and fermentation) would complicate the model, but lead to a similar result.

In this model, the HCO_3_^−^ production rate at distance *z* from the top of a sediment column is given by

*P(z)* *=* *P*_max_(1 − *c(z)/c*_A-min_) (2)

where *P*_max_ is the maximum HCO_3_^−^ production rate (which occurs at the top of the sediment column), *c(z)* is the HCO_3_^−^ concentration at depth *z*, and *c*_A-min_ is the HCO_3_^−^ concentration above which *A* drops below *A*_minimum_ and energy-yielding HCO_3_^−^ production stops. Balancing diffusive transport with reaction rate parameterized in this way leads to the expectation that catabolic reaction rate decreases exponentially with sediment depth.

This relationship between catabolic rate and diffusive distance is consistent with the generally observed strong and initially rapid decline in organic oxidation rate with sediment depth^17^. It is also consistent with the generally strong correlation between organic burial efficiency and sedimentation rate^65,75^. In the extreme case of fast-accumulating organic-rich sediment, sedimentary communities are buried faster than reactants (such as O_2_, NO_3_^−^ and SO_4_^2−^ from the overlying ocean) can diffuse to them and faster than terminal reaction products (such as DIC and CH_4_) can diffuse to a sink (such as the anaerobic methane-oxidizing zone or the seafloor). In this system, abundant and compositionally diverse organic matter may persist in deep sediment because reaction rates are too low to consume it.

## Methods

### Calculation of habitable basement volume

The total volume (rock + water) of submarine igneous basement cooler than 122 °C is 3.0 × 10^8^ km^3^. We calculated this total volume using the method of Heberling et al.^97^; however, instead of globally averaged theoretical and observed heat flow values^98^ and constant sedimentation rate of 3 m Myr^−1^, we used global grids of heat flow^99^ and sediment thickness^100^ to calculate global maps of depth to the 122 °C isotherm with a grid resolution of 0.1 × 0.1 degrees. We then spatially integrated these depths to calculate a global volume.

Assuming 10% in upper 500 m of basement and a linear decrease in porosity to zero at 1000 m into basement^101^, habitable pore space in the basement is 2.2 × 10^7^ km^3^ (about 2% of ocean volume).

### Determination of dissolved O_2_ and SO_4_^2−^ distributions

We used interstitial water concentrations of dissolved O_2_ and SO_4_^2−^ from ODP and IODP drill sites^102^ and a two-step approach to create the global map of electron acceptor distributions in sediment (Fig. 1). The first step was to plot the chemical species as a function of sediment depth and note whether their concentration goes to zero within the sediment or is non-zero all the way to basement. Of 380 ODP and IODP drill sites with SO_4_^2−^ data, SO_4_^2−^ concentrations go to zero within the sediment at 150 sites, 70 sites had non-zero SO_4_^2−^ concentrations from seafloor to basement, and 160 sites were undetermined because the sites were not drilled to basement or SO_4_^2−^ measurements were stopped at depths far above basement. The dissolved O_2_ results were previously reported in D’Hondt et al. (2015)^16^.

**Fig. 5:**
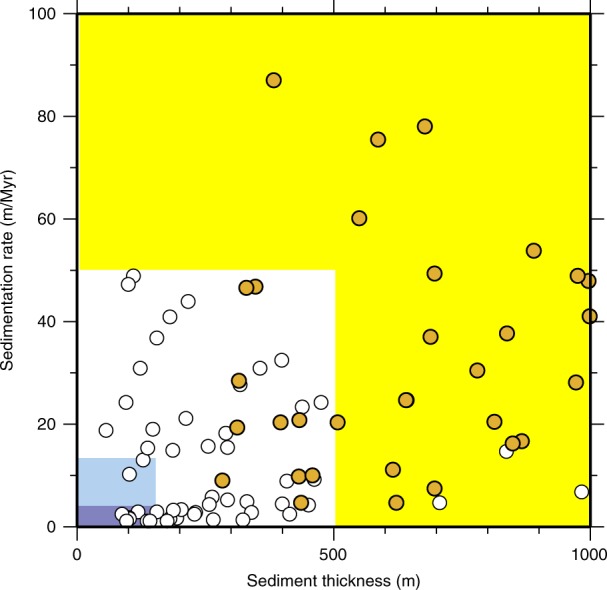
Plot of sediment thickness and sedimentation rate at ODP and IODP sites. Only sites where dissolved SO_4_^2−^ has been measured in sedimentary porewater are shown. Orange dots indicate sites where SO_4_^2−^ concentrations go to zero within the upper 500 m of sediment. White dots indicate sites where SO_4_^2−^ is present from seafloor to basement. The yellow, dark blue and light blue fields identify the sediment thickness and sedimentation rate boundaries used to generate Fig. 1. The blue fields are based on previous results for dissolved O_2_ reported by D’Hondt et al.^16^. Dissolved O_2_ may or may not be present throughout the sediment at sites marked by white dots in the blue fields; no O_2_ measurements were made at these sites. White dots in the yellow areas include sites where flow introduces SO_4_^2−^ through rubble or from beneath the seafloor and sites where SO_4_^2−^ diffuses upward from underlying brine. Orange dots in the white field generally mark sites where dissolved chemical concentrations are not at diffusive steady state (e.g., because sedimentation rate has been unusually high in the recent geologic past)

The second step identified relationships between the presence and absence of the various chemical species as a function of sedimentation rate and total sediment thickness. This method was previously used for O_2_ in sediment;^16^ we extended it to include the SO_4_^2−^ data (Fig. 5). Our results indicate that SO_4_^2−^ goes to zero in the sediment column when sediment thickness exceeds about 500 m or sedimentation rate exceeds 35 m Myr^−1^. In comparison, dissolved O_2_ concentrations go to zero in the sediment where sediment thickness exceeds about 150 m or sedimentation rate exceeds 15 m Myr^−1^. These ‘boundaries’ for persistence or absence of SO_4_^2−^ to igneous basement were combined with similar boundaries for dissolved O_2_ (e.g., D’Hondt et al., 2015) to generate Fig. 1 from global sediment-thickness maps^100^ and depth-averaged sedimentation rates. The depth-averaged sedimentation rates were derived from the sediment thickness maps and global maps of ocean basement age^103^.

### Reaction-rate calculations

To quantify net rates of O_2_ reduction (IODP Site U1370) and SO_4_^2−^ reduction (ODP Sites 1226 and 984) from dissolved chemical data (Fig. 2a–c), we used a modified version of the Matlab-based numerical procedures of Wang et al.^71^. We modified the approach of Wang et al. by using an Akima spline, instead of a 5-point running mean, in order to generate a best-fit line to the chemical concentration data. We determined standard deviations through use of a Monte Carlo simulation (*n* = 50). For these calculations, diffusivities are from Schulz^104^, corrected for in situ temperature at Sites 1226 and U1370 (temperature was assumed constant at Site 984, where in situ temperature was not measured). Porosities and in situ temperature data are from shipboard measurements of the respective drilling expeditions^105–107^ (available at http://sedis.iodp.org). Sedimentation rates are from the shipboard age models generated for each drill site^105–107^.

### Calculation of electron-equivalent burial rates

We calculate electron-equivalent burial rates by multiplying the difference between the dominant oxidation state of the chemical element (C, N or S) at Earth’s surface and its oxidation state in marine sediment. The dominant surface-Earth oxidation states for C, N, and S are IV, 0 and VI, respectively. The oxidation states in marine sediment are taken as 0, -III and -I for organic C, organic N and S in FeS_2_.

### Calculation of alkalinity effect on CO_2_ partial pressure

Increasing alkalinity from present ocean values will cause net conversion of dissolved CO_2_ to predominantly HCO_3_^−^ and to a lesser extent CO_3_^2−^ (dissolved CO_2_ = H_2_CO_3_ + CO_2_). We calculated the exact change in equilibrium CO_2_ partial pressure for a given change in alkalinity using the thermodynamic constants for the aqueous CO_2_ system^49^.

### Dependence of organic degradation on diffusive distance

Our idealized model of the depth dependence of organic degradation rate assumes that the rate of a catabolic reaction sequence (i.e., production of a sugar monomer from particulate organic matter, followed by fermentation and respiration), occurs at or near steady state at a given sediment depth and that the energy (*−ΔG*) yielded by each biologically conserved reaction (fermentation or respiration) must equal or exceed the minimum amount that can be biologically conserved (*ΔG*_minimum_)^77,108,109^. That is, the affinity (*A* = *–ΔG*) for each catabolic reaction must equal or exceed the biological minimum affinity (*A*_minimum_). This assumption is supported by observations of in situ energies of reactions in marine sediment^44,77,110,111^. Here, we use SO_4_^2−^ reduction as an example. In this approach, the catabolic production rate of DIC (of which HCO_3_^−^ is the major species) depends on the HCO_3_^−^ concentration, 1 – *c/c*_*A*-min_, to keep *A ≥ A*_minimum_, where *c*_*A*-min_ is the HCO_3_^−^ concentration above which *A* drops below *A*_minimum_ and the reaction sequence does not proceed. This dependence of rate on metabolite concentrations was previously applied by Boudart (1978)^112^ to generic reaction sequences and adopted by Jin and Bethke (2009)^78^, and Dale et al. (2008)^113^. We extend this approach by explicitly identifying the effect of diffusive distance on reaction rate, via its effect on metabolite concentrations. We choose HCO_3_^−^ for this demonstration because, in sulfate-reducing sediment where sulfide is scavenged by iron, HCO_3_^−^ exhibits the largest relative change in concentration.

*P(z)*, the production of DIC at depth *z* is then given by

2 $$P\left( z \right) = P_{\mathrm{max}}\left( {1 - c\left( z \right)} \right]/\left. {c_{A - {\mathrm{min}}}} \right)$$

where *c(z)* is the HCO_3_^−^ concentration at depth *z*, and *P*_*max*_ is the rate when *c* = 0 and the local mass balance is

3 $$\frac{{\partial c\left( {z,t} \right)}}{{\partial t}} = 0 = D\frac{{\partial ^2c\left( {z,t} \right)}}{{\partial z^2}} + P\left( z \right)$$

4 $$0 = D\frac{{\partial ^2c(z,t)}}{{\partial z^2}} + P_{\mathrm{max}}\left(1 - \frac{{c\left( z \right)}}{{c\left( z \right)_{A - {\mathrm{min}}}}}\right)$$

where *D* is the HCO_3_^−^ diffusion constant. A solution to Eq. (3) is

$$c\left( z \right) = \left( {c\left( {z = 0} \right)-c_{A - {\mathrm{min}}}} \right) \ast \left( {{\mathrm{exp}}\left( { - P_{\mathrm{max}}} \right./\left( {D \ast c_{A - {\mathrm{min}}}} \right.} \right) + c_{A - {\mathrm{min}}}$$

and the depth-dependent metabolic rate is then

$$P\left( z \right) = P_{\mathrm{max}} \ast {\mathrm{exp}}\left( {\left( { - P_{\mathrm{max}}/\left( {Dc_{A - {\mathrm{min}}}} \right)^{0.5}z} \right)} \right)$$

Where *P*_max_ = DIC production rate when *c*_*DIC*_ = 0.

### Turnover-time calculations

The turnover times of low-molecular-weight fatty acids are calculated as the concentration of lactate, acetate or formate divided by the rate of SO_4_^2−^ reduction corrected for stoichiometry (respectively 1.0, 0.67, and 0.5 times the rate of SO_4_^2−^ reduction for acetate, lactate, and formate) at IODP Site 1226, a site where SO_4_^2−^ is reduced but not entirely depleted. The different ratios to SO_4_^2−^ reduction are used to account for the different oxidation states and numbers of carbon atoms in each molecule, assuming the initial particulate organic carbon (POC) had oxidation state 0. Because we do not know the fraction of total SO_4_^2−^ reduction rate that goes to oxidize each of these low-molecular-weight acids, we calculated a potential range of turnover times based on each one separately. At this site, concentrations of these low-molecular-weight fatty acids are all in the range of 1 µM^111^ and the subseafloor SO_4_^2−^ reduction rate declines from 3 × 10^−8^ moles liter^−1^ yr^−1^ to ~0.2 × 10^−8^ moles liter^−1^ yr^−1^ with increasing depth below seafloor^71^. Given the relatively constant low-molecular-weight fatty acid concentrations, the decline in SO_4_^2−^ reduction rate leads to turnover times increasing from the low tens to low hundreds of years with increasing sediment depth.

## Data Availability

All data are previously published and accessible as identified in the cited sources.
